# Présentation atypique d'une granulomatose avec polyangeite: à propos d'une observation pédiatrique

**DOI:** 10.11604/pamj.2015.21.141.7086

**Published:** 2015-06-22

**Authors:** Olfa Berriche, Samia Younes, Wafa Ammari, Wafa Alaya, Wassia Kessomtini, Sonia Hammami

**Affiliations:** 1 Service de Médecine Interne, Hopital Taher Sfar, Mahdia, Tunisie; 2 Service d'Ophtalmologie, Hopital, Taher Sfar, Mahdia, Tunisie; 3 Unité de Médecine Physique, Hopital Taher Sfar, Mahdia, Tunisie; 4 Service de Médecine Interne, Hopital Fattouma Bourguiba, Monastir, Tunisie

**Keywords:** Granulomatose, enfant, vascularite, Granulomatosis, child, vasculitis

## Abstract

La granulomatose avec polyangéite (GPA) est une vascularite nécrosante systémique, caractérisée par une inflammation granulomateuse, une nécrose tissulaire et une vascularite touchant les vaisseaux de moyen et, surtout, de petit calibre, elle touche rarement l'enfant.

## Introduction

La GPA fait partie des vascularites systémiques touchant les vaisseaux de petit calibre, selon la classification adoptée lors de la conférence de consensus de Chapel Hill. L’âge moyen d'entrée dans la GPA est généralement entre 35 et 55 ans [1]. Les formes pédiatriques, plus rares, partagent certaines caractéristiques avec la forme de l'adulte mais présentent certaines particularités. Nous rapportons l'observation d'une enfant âgée de sept ans dont la maladie a été inaugurée par une symptomatologie oculaire et des signes généraux non spécifiques. Le diagnostic de GPA était suspecté, sept ans après, devant une symptomatologie digestive et ORL. Seuls quelques cas pédiatriques avec une présentation inhabituelle ont été rapportés dans la littérature, le diagnostic doit toutefois être posé rapidement pour diminuer la morbi-mortalité liée à la maladie.

## Patient et observation

Une Patiente âgée de 7 ans, hospitalisée à plusieurs reprises pour une altération de l´état général, et une tuméfaction palpébrale persistante. L'enquête étiologique initiale était négative. L’évolution ultérieure sept ans après, était marquée par de douleurs abdominales diffuses récidivantes dans un contexte d'apyrexie, des polyarthralgies de type inflammatoire des grosses articulations, un flou visuel et une rhinorrhée bilatérale. L'examen ORL mettait en évidence une ensellure nasale, et une formation polyploïde de la fosse nasale gauche. L'examen ophtalmologique trouvait une dacryoadénite droite avec une kératite filamenteuse. L´hémogramme montrait une anémie hypochrome microcytaire (Hb à 8.7 g/dl, TCMH à 23pg VGM à 70fl,). La vitesse de sédimentation était à 80mm Hg à la première heure, La fibrinémie était à 4g/L et l´électrophorèse des protides montrait une albuminémie à 23 g/L. Le bilan rénal montrait une créatinine à 59µmol/l, avec une protéinurie de 24h à 1,4g/24h et une hématurie microscopique. La sérologie syphilitique, la recherche de mycobactéries dans les crachats et les urines ainsi que l'intra dermo réaction étaient négatives. Les anticorps anti nucléaires (AAN) étaient négatifs et les anticorps dirigés contre le cytoplasme des polynucléaires neutrophiles, à renforcement cytoplasmique en immunofluorescence indirecte (c-ANCA) et de spécificité´ anti protéinase 3 (anticorps anti-PR3), étaient positifs à 1/1200.

**Figure 1:**
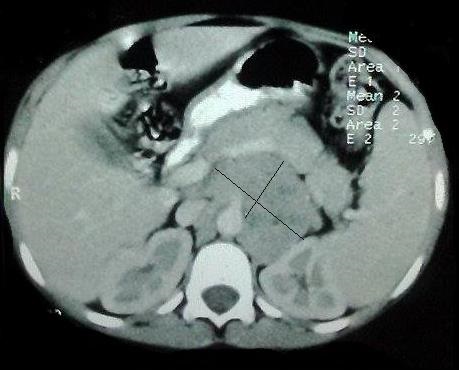
TDM abdomino pelvienne en coupe axiale montrant une masse tissulaire retro péritonéale gauche faisant 8x5 cm à contours bosselé en rapport avec un amas ganglionnaire

La biopsie des glandes lacrymales a objectivé une inflammation chronique granulomateuse non nécrosante. La TDM du massif facial a objectivé une hypoplasie bilatérale des sinus maxillaires avec un épaississement polypoïde ainsi qu'une obstruction des deux méats moyens. La radiographie thoracique était normale. Une TDM abdominopelvienne objectivait une masse tissulaire retro péritonéale gauche faisant 8x5 cm à contours bosselé en rapport avec un amas ganglionnaire (Figure 1). La ponction ganglionnaire a ramené un matériel hémorragique sans cellules suspectes de malignité.

La biopsie rénale a objectivé une hyalinose segmentaire et focale avec des lésions de vascularite. Le diagnostic de granulomatose avec polyangéite (GPA) était retenu devant la présence de 3 critères de l'ACR: l'atteinte ORL, l'hématurie microscopique, l'inflammation granulomatose à la biopsie lacrymale.

La patiente était mise sous corticothérapie à la dose de 2mg/kg/j pendant 1mois avec une dégression progressive, elle a reçu 8 cures de cyclophosphamide sur 6 mois à la dose de 600mg/m^2^ et une antibiothérapie par Bactrim^®^ à la dose de 2cp/j, la patiente était par la suite perdue de vue pendant 7 ans. La patiente a consulté par la suite pour asthénie et récidive des douleurs abdominales. A la biologie: le bilan rénal montrait: une protéinurie de 24H à 6g, une hématurie microscopique et une créatinine à 70mmol/l. La ponction biopsie rénale montrait le même aspect initial.

Le scanner du massif facial de contrôle a montré un épaississement en cadre du sinus maxillaire droit, une déminéralisation de l'os maxillaire et un épaississement du sinus sphénoïdal gauche. Le scanner abdominal de contrôle a objectivé la persistance de la masse ganglionnaire retro péritonéale qui a légèrement régressé en taille avec apparition des foyers centraux nécrosés.

Un traitement par corticothérapie à base de méthylprednisolone à la dose de 15 mg/kg/j a été débuté pendant 3 jours, relayé par une corticothérapie orale à la dose de 1mg/kg/j pendant un mois, avec diminution progressive des doses, associée à des bolus bimensuels de cyclophosphamide en intraveineux à la dose de 600 mg/m^2^ à J0, J14 et J28 puis à la dose de 0,7mg/m^2^ tous les 21 jours.

La patiente a reçu au total 6 bolus de cyclophosphamide relayés par l'azathioprine (Imurel^®^) à la dose de 2 mg/kg/j, du cotrimoxazole à la dose de 2cp/j était également prescrit. L’évolution était marquée par une amélioration des signes cliniques et une négativation de la protéinurie de 24 h au bout de 4 mois. Le recul actuel est de 6 mois.

## Discussion

La granulomatose avec poly angéite est une vascularite nécrosante des petits vaisseaux associant une inflammation de la paroi vasculaire et une granulomatose, péri- et extravasculaire. Elle peut se présenter cliniquement sous une forme diffuse, systémique d'emblée, ou sous une forme localisée. Ses manifestations les plus caractéristiques sont les atteintes des sinus, des poumons et des reins mais il existe d'autres manifestations beaucoup plus rares, parmi lesquelles figurent les complications ophtalmologiques [2] et digestives [3].

Les formes pédiatriques sont rares et très peu de données épidémiologiques et cliniques sont disponibles du fait du faible effectif de patients concernés, leur prise en charge est souvent difficile et plus délicate que chez l'adulte [4]. La maladie débute à l'adolescence d'après la majorité des séries rapportées [5, 6], un début plus précoce, en moyenne vers l’âge de sept ans comme c'est le cas chez notre patiente n'a été que rarement rapporté [7]. La présentation clinique est souvent plus atypique que chez l'adulte et marquée par une longue latence entre présentation clinique et diagnostic, et ceci d'autant plus que les ANCA peuvent être négatifs initialement.

Dans notre observation il s'agissait d'une enfant âgée de 7 ans dont la maladie était inaugurée par un tableau atypique fait par l'association de signes généraux et d'une atteinte oculaire à type de dacryoadénite avec à l'histologie une inflammation chronique granulomateuse non nécrosante. La fréquence de l'atteinte oculaire dans GPA varie entre 28 et 87% des patients, selon les séries [8, 9]. Son incidence est estimée entre 10 et 50% chez l'enfant. Elle peut se manifester par une conjonctivite, une sclérite ou épisclérite, une dacryocystite, ou plus fréquemment une inflammation orbitaire, Souvent, c'est une atteinte initialement isolée précurseur de la maladie et il s'agit le plus souvent d'une atteinte réfractaire au traitement [10]. L'atteinte oculaire est inaugurale dans 10 à 23% des cas [11], et peut parfois en rester une forme limitée longtemps isolée. Mais, assez souvent, elle apparaitprécocement ou survient lors d'une poussée évolutiveau sein d'autres manifestations systémiques parfois sévères.

Dans notre observation le diagnostic de GPA était suspecté, sept ans après le début des symptômes, devant l'association secondaire de signes digestifs et ORL non spécifiques. Le tableau mimait une néoplasie métastatique ou un lymphome devant la présence d'adénopathies abdominales au scanner abdominal, ce qui rendait le diagnostic plus difficile. Ces aspects trompeurs ne doivent pas exclure le diagnostic mais posent souvent le problème des diagnostics différentiels. Notre patiente présentait également une atteinte rénale, cette atteinte est plus rare que chez l'adulte mais d’évolution similaire et elle est classiquement plus fréquente et plus sévère dans la polyangéitemicroscopique [12].

La GPA s'associe à la présence d'anticorps anti-cytoplasme des polynucléaires(ANCA), auto-anticorps dirigés contre deux antigènes présents dans le cytoplasme des polynucléaires neutrophiles. La prévalence des ANCA est variablement estimée dans les séries pédiatriques; ils sont trouvés chez 82% des enfants remplissant les critères de l'ACR de la série de Belostosky et chez 95,5% des patients de la série canadienne avec une spécificité anti protéinase 3 dans 80% des cas [5, 7]. L'atteinte rénale et digestive a été précédée chez notre patiente par un tableau d'atteinte oculaire associée à des signes généraux, cette succession a été rarement décrite dans la littérature.

Le traitement de la GW de l'enfant n'est pas encore bien codifié. Par analogie à l'adulte, le cyclophosphamide, par voie orale ou mieux par voie intraveineuse en association avec la corticothérapie est le traitement de référence au cours des poussées [13].

## Conclusion

Notre observation illustre une présentation atypique mais sévère de la GPA, La rechute a l'arrêt du traitement laisse prédire une forme à dominante granulomateuse exposant a des rechutes fréquentes. Chez un enfant présentant une atteinte oculaire trainante d'origine indéterminée, ou une présentation digestive pseudo tumorale doivent constituer des signes d'appel pour orienter en urgence le diagnostic surtout devant lamorbi-mortalité importante de la maladie et la nécessité d'une prise en charge immédiate.
